# Source control in emergency general surgery: WSES, GAIS, SIS-E, SIS-A guidelines

**DOI:** 10.1186/s13017-023-00509-4

**Published:** 2023-07-21

**Authors:** Federico Coccolini, Massimo Sartelli, Robert Sawyer, Kemal Rasa, Bruno Viaggi, Fikri Abu-Zidan, Kjetil Soreide, Timothy Hardcastle, Deepak Gupta, Cino Bendinelli, Marco Ceresoli, Vishal G. Shelat, Richard ten Broek, Gian Luca Baiocchi, Ernest E. Moore, Ibrahima Sall, Mauro Podda, Luigi Bonavina, Igor A. Kryvoruchko, Philip Stahel, Kenji Inaba, Philippe Montravers, Boris Sakakushev, Gabriele Sganga, Paolo Ballestracci, Manu L. N. G. Malbrain, Jean-Louis Vincent, Manos Pikoulis, Solomon Gurmu Beka, Krstina Doklestic, Massimo Chiarugi, Marco Falcone, Elena Bignami, Viktor Reva, Zaza Demetrashvili, Salomone Di Saverio, Matti Tolonen, Pradeep Navsaria, Miklosh Bala, Zsolt Balogh, Andrey Litvin, Andreas Hecker, Imtiaz Wani, Andreas Fette, Belinda De Simone, Rao Ivatury, Edoardo Picetti, Vladimir Khokha, Edward Tan, Chad Ball, Carlo Tascini, Yunfeng Cui, Raul Coimbra, Michael Kelly, Costanza Martino, Vanni Agnoletti, Marja A. Boermeester, Nicola De’Angelis, Mircea Chirica, Walt L. Biffl, Luca Ansaloni, Yoram Kluger, Fausto Catena, Andrew W. Kirkpatrick

**Affiliations:** 1 General, Emergency and Trauma Surgery Dept., Pisa University Hospital, Via Paradisia, 56124 Pisa, Italy; 2 General Surgery Dept., Macerata Hospital, Macerata, Italy; 3 Department of Surgery, Western Michigan University Homer Stryker MD School of Medicine, Kalamazoo, MI USA; 4 Anadolu Medical Center, Kocaeli, Turkey; 5 ICU Dept., Careggi University Hospital, Florence, Italy; 6 Department of Surgery, College of Medicine and Health Sciences, United Arab Emirates University, Al-Ain, United Arab Emirates; 7 Department of Gastrointestinal Surgery, Stavanger University Hospital, University of Bergen, Bergen, Norway; 8 Dept. of Health – KwaZulu-Natal, Surgery, University of KwaZulu-Natal and Inkosi Albert Luthuli Central Hospital, Durban, South Africa; 9 All India Institute of Medical Sciences, New Delhi, India; 10 Department of Surgery, John Hunter Hospital, Newcastle, Australia; 11 General Surgery Dept., Monza University Hospital, Monza, Italy; 12 Department of General Surgery, Tan Tock Seng Hospital, Singapore, Singapore; 13 Department of Surgery, Radboud University Medical Center, Njmegen, The Netherlands; 14 General Surgery Dept., Brescia University Hospital, Brescia, Italy; 15 Surgery Dept., Denver Trauma Centre, Denver, CO USA; 16 Département de Chirurgie, Hôpital Principal de Dakar, Hôpital d’Instruction des Armées, Dakar, Senegal; 17 Department of Surgical Science, University of Cagliari, Cagliari, Italy; 18 General Surgery, San Donato Hospital, Milan, Italy; 19 Department of Surgery No. 2, Kharkiv National Medical University, Kharkiv, Ukraine; 20 Department of Surgery, East Carolina University, Brody School of Medicine, Greenville, NC USA; 21 LAC+USC Medical Center, Los Angeles, CA USA; 22 Département d’Anesthésie-Réanimation CHU Bichat Claude Bernard, Paris, France; 23 Research Institute of Medical, University Plovdiv/University Hospital St. George, Plovdiv, Bulgaria; 24 Fondazione Policlinico Universitario A. Gemelli IRCCS, Università Cattolica del Sacro Cuore, Rome, Italy; 25 First Department of Anaesthesiology and Intensive Therapy, Medical University of Lublin, Lublin, Poland; 26 Dept. of Intensive Care, Erasme Univ Hospital, Brussels, Belgium; 27 General Surgery, Hospital, National and Kapodistrian University of Athens (NKUA), Athens, Greece; 28 University of Otago, Otago, New Zealand; 29 Clinic of Emergency Surgery, University Clinical Center of Serbia, Belgrade, Serbia; 30 Infectious Disease Dept., Pisa University Hospital, Pisa, Italy; 31 Anesthesiology, Critical Care and Pain Medicine Division, Department of Medicine and Surgery, University of Parma, Parma, Italy; 32 Department of War Surgery, Kirov Military Medical Academy, Saint-Petersburg, Russia; 33 Surgery Department, Tbilisi State Medical University, Tbilisi, Georgia; 34 General Surgery Dept, San Benedetto del Tronto Hospital, San Benedetto del Tronto, Italy; 35 Emergency Surgery, Meilahti Tower Hospital, Helsinki, Finland; 36 Groote Schuur Hospital and University of Cape Town, Cape Town, South Africa; 37 Trauma and Acute Care Surgery Unit, Hadassah - Hebrew University Medical Center, Jerusalem, Israel; 38 Department of Traumatology, John Hunter Hospital and University of Newcastle, Newcastle, NSW Australia; 39 Department of Surgical Disciplines, Immanuel Kant Baltic Federal University, Regional Clinical Hospital, Kaliningrad, Russia; 40 University Hospital of Giessen, Giessen, Germany; 41 Government Gousia Hospital, Srinagar, Kashmir India; 42 PS-SS, Weissach im Tal, Germany; 43 Department of Emergency Surgery, Centre Hospitalier Intercommunal de Villeneuve-Saint-Georges, Villeneuve-Saint-Georges, France; 44 Virginia Commonwealth University, Richmond, VA USA; 45 ICU Dept., Parma University Hospital, Parma, Italy; 46 General Surgery Dept., Mozir Hospital, Mozir, Belarus; 47 Emergency Department, Radboud University Medical Center, Njmegen, The Netherlands; 48 Trauma and Acute Care Surgery, Foothills Medical Center, Calgary, AB Canada; 49 Infectious Disease Dept., Udine University Hospital, Udine, Italy; 50 Tianjin Nankai Hospital, Tianjin Medical University, Tianjin, China; 51 Riverside University Health System Medical Center, Riverside, CA USA; 52 Loma Linda University School of Medicine, Loma Linda, CA USA; 53 Department of General Surgery, Albury Hospital, Albury, Australia; 54 ICU Dept., Bufalini Hospital, Cesena, Italy; 55 Surgery Dept., Amsterdam University, Amsterdam, The Netherlands; 56 Service de Chirurgie Digestive et Hépato-Bilio-Pancréatique, Hôpital Henri Mondor, Université Paris Est, Créteil, France; 57 Centre Hospitalier Universitaire Grenoble Alpes, Grenoble, France; 58 Trauma and Emergency Surgery, Scripss Memorial Hospital, La Jolla, CA USA; 59 General Surgery, Pavia University Hospital, Pavia, Italy; 60 General Surgery, Rambam Medical Centre, Haifa, Israel; 61 General, Emergency and Trauma Surgery Dept, Bufalini Hospital, Cesena, Italy; 62 General, Acute Care, Abdominal Wall Reconstruction, and Trauma Surgery, Foothills Medical Centre, Calgary, AB Canada

**Keywords:** Source control, Emergency, Infections, Abdominal, Surgery, Trauma, Mortality, Antibiotic, Stewardship

## Abstract

Intra-abdominal infections (IAI) are among the most common global healthcare challenges and they are usually precipitated by disruption to the gastrointestinal (GI) tract. Their successful management typically requires intensive resource utilization, and despite the best therapies, morbidity and mortality remain high. One of the main issues required to appropriately treat IAI that differs from the other etiologies of sepsis is the frequent requirement to provide physical source control. Fortunately, dramatic advances have been made in this aspect of treatment. Historically, source control was left to surgeons only. With new technologies non-surgical less invasive interventional procedures have been introduced. Alternatively, in addition to formal surgery open abdomen techniques have long been proposed as aiding source control in severe intra-abdominal sepsis. It is ironic that while a lack or even delay regarding source control clearly associates with death, it is a concept that remains poorly described. For example, no conclusive definition of source control technique or even adequacy has been universally accepted. Practically, source control involves a complex definition encompassing several factors including the causative event, source of infection bacteria, local bacterial flora, patient condition, and his/her eventual comorbidities. With greater understanding of the systemic pathobiology of sepsis and the profound implications of the human microbiome, adequate source control is no longer only a surgical issue but one that requires a multidisciplinary, multimodality approach. Thus, while any breach in the GI tract must be controlled, source control should also attempt to control the generation and propagation of the systemic biomediators and dysbiotic influences on the microbiome that perpetuate multi-system organ failure and death. Given these increased complexities, the present paper represents the current opinions and recommendations for future research of the World Society of Emergency Surgery, of the Global Alliance for Infections in Surgery of Surgical Infection Society Europe and Surgical Infection Society America regarding the concepts and operational adequacy of source control in intra-abdominal infections.

## Background

Intra-abdominal infections (IAIs) are an important global cause of morbidity and mortality and one of the main etiologies of sepsis [1]. Intra-abdominal sepsis (IAS) is a severe medical/surgical emergency that affects the entire body, still in 2022 has dismal outcomes, and is misunderstood and generally poorly appreciated by the public and medical professionals alike [2]. Out of a Global burden of 50 million septic cases and 11 million sepsis-related deaths worldwide [3], IAS constitutes the second leading cause. Furthermore, it appears to be the type of sepsis least understood by physicians globally. Anatomically, IAS also presents unique challenges. The abdomen represents a reservoir of a massive microbial population contained with the human microbiome, that controls all human health, but is extremely sensitive to shock and stress. Further, both the bacterial flora and the health of the GI tract itself are profoundly influenced by several factors including intra-abdominal pressure, shock, and malperfusion.

The actual definition of sepsis was recently refined to emphasize systemic pathobiology emphasizing sepsis should be defined as life-threatening organ dysfunction caused by a dysregulated host response to infection, and that any sepsis is severe and in particular, septic shock [1]. However, concerns have been raised from this new definition of sepsis oversimplify the pathophysiologic progression involving a typical patient, and that this paradoxically now complicates the clinical management of any individual patient. The preceding accepted definition had stratified a few functional steps describing a progression from systemic inflammation up to septic shock passing through sepsis and severe sepsis. This allowed clinicians to practically stratify patients in risk/benefit categories regarding interventions with potential serious side effects and iatrogenic morbidity. This working stratification is especially relevant in best managing IAS, as potential therapies range from noninvasive antibiotic therapy only, to minimally invasive approaches, and finally open surgical techniques with sometimes permanent anatomic changes. For instance, whereas a laparotomy and defunctioning colostomy is not typically required for a sigmoid diverticular micro perforation in a patient with only fever and tachycardia, it is in a patient with vasomotor shock and progressive organ failure not responding to simpler methods. Thus, the combination and timing of different physical techniques are strongly influenced by the conditions of the patients that are better reflected by the previous sepsis definition [4]. Data from the Global WISS study noted a 41% mortality rate [5, 6], and in the developing world mortality rates may be 80% with septic shock [7]. Of those who die, most are from multiple organ failure which is a still poorly understood consequence rather than the immediate effect of infection. This reflects the sequelae of a local problem becoming systemic, with a dysregulated host response and progressive organ failure because of the elaboration and systemic propagation of inflammatory biomediators [8, 9].

## Standard therapy of severe intra-abdominal sepsis

Early recognition of the patient with ongoing IAS is an essential step for an effective treatment. Prompt administration of empiric broad-spectrum antibiotic therapy (AT) and judicious intravenous fluids for resuscitation are crucial. Potential adjuncts and amelioration or at least modulation of systemic inflammation may contribute to improve final outcomes [10]. This initial resuscitation should be titrated to the clinical response, and not solely guided by a predetermined protocol. Vasopressor agents may serve to augment and assist fluid resuscitation. It is debated if the source control measures should only be undertaken once the patient has been appropriately stabilized, although resuscitation should proceed as rapidly as feasible [11, 12]. In the most severe cases, it may be appropriate to proceed with invasive source control even while ongoing resuscitation is continuing as the risk is the patient will die before than can be otherwise “optimized” for surgery.

## Source control of intra-abdominal sepsis

Source control (SC) is an essential element in IAI management. Delay in providing adequate SC has been associated with adverse outcomes including death in IAS [13–16]. Any individual patients’ actual surgical condition(s), his/her comorbidities, and previously administered therapies combined to the source of infections and the timing of presentation must be considered together and all-important parts of the decision-making process to plan the best diagnostic-therapeutical strategy. Antibiotic therapy represents an indissociable part of a correct infection management. Extra-abdominal sepsis, however, may be successfully treated with the only antibiotic therapy, abdominal sepsis not. However, despite accepted by all as “necessary” the actual definition and practical application of SC are still debated. Many studies use the term “appropriate or adequate source control” in association with a patient’s clinical improvement or to justify duration of antibiotic therapy. However, without a universally agree upon and unequivocal definition of general and above all of SC adequacy, such guidelines and indications are functionally impossible to apply without bias or confusion.

Therefore, the timing, involved strategies, the adequacy, and the ultimate results of SC may vary between patients and different clinical scenarios.

Therefore, given the extreme importance of providing timely and appropriate SC in critically ill patients with IAS, the aim of the present paper is to define SC, its adequacy and appropriateness in the different abdominal emergency general surgical conditions.

### Notes on the use of the guidelines

The practice promulgated in this work does not represent a clearly accepted standard of practice. Concepts and approaches described are suggested plans of care, based on best available evidence and the consensus of experts, but they do not exclude other approaches as being within the standard of practice. For example, they should not be used to compel adherence to a given method of medical management, which method should be finally determined after taking account of the conditions at the relevant medical institution (staff levels, experience, equipment, etc.) and the characteristics of the individual patient. However, responsibility for the results of treatment rests with those who are directly engaged therein, and not with the consensus group. Given the paucity of high-level scientific evidence, it is also the hope of the authors that these guidelines will be revised regularly as new evidence becomes available to incorporate into guidelines development.

## Methods

A computerized search was done by the bibliographer in different databanks (MEDLINE, SCOPUS, EMBASE) citations were included for the period between January 1980 to May 2022 using the primary search strategy: intra-abdominal, infections, hemodynamic instability/stability, management, source control, surgical, radiological, antibiotic, therapy, damage control, combined with AND/OR. No search restrictions were imposed. The dates were selected to allow comprehensive published abstracts of clinical trials, consensus conference, comparative studies, congresses, guidelines, government publication, multicenter studies, systematic reviews, meta-analysis, large case series, original articles, randomized controlled trials. Case reports and small cases series were excluded. Narrative review articles were also analyzed to determine other possible studies. A group of experts in the field coordinated by a central coordinator was contacted to express their evidence-based opinion the issues. Through subsequent rounds, the different topics were discussed, and the paper implemented. The final version about which the agreement was reached consisted in present paper.

### Anatomic, pathophysiologic, and pathobiological determinants of IAS

While practically the anatomy, physiology, pathology including pathobiology of IAS are inexorably linked in innumerable loops and interconnections, they are discussed separately for ease of understanding.

#### Anatomic

The abdominal cavity is a semirigid container with a finite volume that is subject to the laws of hydrostatics. When the intra-abdominal volume increases due to visceral swelling, intra-peritoneal leakage or hematoma, or for any other reason; intra-abdominal pressure (IAP) rises on an asymptotic basis [17]. Practically, this makes abnormally high IAP, known as intra-abdominal hypertension (IAH) a ubiquitous feature of critical illness/injury. IAH should be equated with visceral ischemia and not ignored [18, 19]. Even if IAH is mild, it still associates with adverse outcomes in critically ill patients [20]. If the IAH is severe enough to acutely and reproducibly induce acute organ failure, the overt abdominal compartment syndrome is diagnosable [21], which entails a mortality from 75.9% to near 100% depending on the urgency of rescue [22].

#### Pathophysiologic

IAS and especially subsequent therapies involving fluid resuscitation are significant risk factors for IAH, ACS, and subsequent multi-system organ failure (MSOF). IAH/ischemia adversely effects the entire body, through both physical and humoral mechanisms. The physical effects are quite well described including cardiovascular collapse, respiratory compromise including worsening pulmonary edema and ARDS, and gastrointestinal, renal, and even central nervous system failure. Less appreciated are the consequences of toxic bio-mediator generation, which drives multi-system organ failure (MSOF), even after source control is achieved [23].

#### Pathobiological

The human microbiome is the collection of all microbes that reside within and upon the human body (such as bacteria, fungi, viruses, and their genes) [8, 24]. There are 150-fold more bacterial genes than our own, in the human microbiome [8, 25]. When subjected to stresses such as septic shock, a healthy microbiome rapidly evolves into a pathologic state, designated a dysbiome. Dysbiosis is defined by the transition to a dysbiome and is a functional change in the intestinal microbiota associated with overgrowth of pathobionts that dramatically alters immune responses [8]. Dysbiosis is marked by the various changes including loss of diversity (predominance of a bacterial group) and decreased richness (decreased number of different species). Both conditions are important in terms of immune response. This loss of microbial diversity reflects gross overrepresentation of pathogenic organisms and decrease of nonpathogenic organisms, that combined with loss of gut barrier integrity yields a greater potential to translocate to extra-intestinal sites. This state potentiates the inflammatory cascade and contributes to multi-system organ failure [25]. Anatomically, all these catastrophic changes occur within the semirigid confines of the abdominal cavity, which can tolerate small volume changes, but becomes exponentially tight inducing catastrophic IAH, with resultant severe ischemia [17, 19]. We have previously made the analogy that unfortunately, evolution has placed the microbiome “time-bomb” within the “pressure cooker” of the abdominal cavity [8].

### Core definitions

#### Peritonitis

Clinicians typically recognize IAS as peritonitis clinically but classify the condition as IAS once managing the patient [26]. Peritonitis is simply any inflammation of the peritoneal lining of the abdominopelvic cavity, with abdominal rigidity is a clinical finding in abdominal palpation. Peritonism is generalized rigidity of the abdomen. While the origins of the peritoneal irritants that result in peritonitis are many, including non-infectious and benign ones, peritonitis may be not infrequently a sign of catastrophe [24]. Peritonitis can be localized or isolated to a certain sector of the abdomen (localized peritonitis) or diffuse with all locations in the abdomen involved (diffuse peritonitis) with the latter being again ominous. With very few exceptions, patients presenting with diffuse peritonitis require immediate surgical exploration, while those with localized clinical signs may be able to undergo further evaluation [24, 27]. Further classic definitions consider primary, secondary, and potentially tertiary etiologies. At all times it should be remembered that while “peritonitis” defines the clinical findings, IAS with progressive organ failure is what kills the patient.

*Primary peritonitis* is defined as spontaneous bacterial seeding of the peritoneal cavity, which typically requires the presence of a bacterial medium within the peritoneal cavity, such as cirrhotic ascites or peritoneal dialysate [24]. *Secondary peritonitis* defines irritation of the abdominal peritoneal lining caused by direct contact with a peritoneal contaminant. It occurs most commonly from a physical or functional disruption of gastrointestinal tract integrity and is typically polymicrobial [24]. *Tertiary peritonitis* is poorly defined, misunderstood, and potentially historical. Upon our review, it was defined most recently in 2005 as *ongoing peritonitis:* “peritonitis that persists or recurs ≥ 48 h following apparently successful management of primary or secondary peritonitis” [28]. Microbiologically, it is associated with a shift from gram negative and enteric bacteria to nosocomial populations. However, it appears that the concepts related to ongoing peritonitis predate the evolving appreciation of the human microbiome and more importantly, the consequences of its devolution to a pathologic dysbiome in critical illness [8, 29, 30]. Finally, a functionally useful set of definitions is the concept of severe and complicated IAS. Severe complicated intra-abdominal sepsis (SCIAS) encompasses one of the most challenging situations surgeons may encounter. IAS is defined as severe when associated with organ dysfunction [6, 31] and as complicated when the inflammation or contamination spreads beyond a single organ, causing either localized or diffuse peritonitis [32].

### Source control definition

The authors propose that “Source control” be understood as the set of all physiological/pharmacological/interventive measures adopted to control a focus of infection, to modify factors in the infectious milieu promoting microbial growth or impair host antimicrobial defenses, and to allow the organism to recover the homeostasis or at least a sort of “physiological equilibrium” (Table 1) [33, 34].

**Table 1:** Adverse physiology and goals of surgical source control

Adverse physiology and goals of surgical source control	
Adverse physiology	Countermeasure from source control
Microbiological (MB) Invasive microbiological infections	Prevent growth of microbiologic organisms
GI Integrity (GIT) Disruption of Gastrointestinal tract integrity	Restore Integrity of the gastrointestinal tract
Biomediator (BioM) Systemic Propagation of Biomediators	Removal, mitigation, or down-regulation of Systemic biomediators
Physiology (Physiol) Systemic elaboration of acid/base electrolyte Imbalance	Restore acid/base electrolyte physiology
Microbiome (MBiome) Evolution from a healthy microbiome to a dysbiome	Minimize the negative selective pressures toward a dysbiome
Intra-abdominal hypertension (IAH/Ischem) Visceral Ischemia resulting from severe Intra-abdominal hypertension	Restore visceral perfusion through treatment of severe Intra-abdominal hypertension

This comprehensive definition includes foremost the removal of all macroscopic gross intra-abdominal contamination and ensuring the cessation of further such contamination.

Further, however, SC must encompass other interconnected and combined actions and interventions including:

- Antibiotic/anti-infective therapy
- Surgery
- Minimally invasive non-surgical/radiological procedures
- Physiological support and restoration aiming to reduce the disease burden

*Antibiotics therapy* has been improved over the years; it represents a fundamental part in managing IAIs. Nowadays the concept of antibiotic stewardship tried to improve the anti-infective drugs management trying to adapt the therapy to the specific patients and their diseases to limit antibiotic resistances [35–37]. A few selected cases of IAIs may be effectively treated with antibiotics alone. Surgical SC and AT are complementary, and an effective and accurate surgical SC may allow to reduce the antibiotic usage, to increase their effectiveness and to positively modify treatment duration [38]. Shorter, targeted antibiotic therapeutic regimens should be advised to reduce the spread of antibiotic resistance. The use of rapid molecular tests might reduce the need for broad-spectrum empiric antibiotic therapy [35, 39]. In some countries, the need for anti-anerobic and anti-ESBL coverage has led to an increased use of carbapenems leading to selection of carbapenemase-producing strains in gram negative enteric pathogens [40]. In all those settings in which resistances are diffused Carbapenem-sparing strategies are therefore desirable [41]. Alternative regimens with carbapenem-sparing beta lactam/beta lactamase inhibitor combinations (BL-BLICs) have been proposed [35, 42] although novel beta-lactams displaying anti-carbapenemase activity should be reserved to patients colonized with CRE o CR-non-fermenting gram negatives or upon rapid identification of genetic determinants using molecular tests, to tailor the use of novel molecules according to the underlying genetic profile [43–46]. Despite the controversies related to the MERINO trial, piperacillin/tazobactam should not be de-prescribed, as post hoc results account for its efficacy in certain contexts [42, 47], to reduce the use of carbapenems, especially in countries with high level of CRE when low inoculum is suspected and for MIC > 4 mg/L. Other novel BL-BLICs can be valid therapeutic tools [48], ceftolozane/tazobactam may preserve its activity against ampC and ESBL producers. Ceftazidime/avibactam with its displayed activity against KPC and OXA-48 should be reserved for these strains. Meropenem/vaborbactam also demonstrated activity against KPC, whereas it retains no activity against OXA-48 producing strains. This said, meropenem also offers anti-anaerobic coverage. Hence, in this setting, metronidazole should be added only when carbapenems are not used [49, 50].

*Surgery* is essentially based on four principles that may be differently combined:

- Drainage
- Debridement associated with dead tissues and/or devices removal
- Decompression
- Restoration of anatomy and function [11].

These principles should be applied according to the causative disease and the patient’s conditions.

In fact, early surgery is not always the best option. The timing of surgery must be considered, together with the best operational and patient management strategy. The three main ways to proceed to surgical source control are: open surgery, laparoscopic surgery, and mini-invasive/radiological procedures.

The principal aim of surgery (open or laparoscopic) is the physical removal of infected or necrotic tissue encompassing several procedures ranging from abscess incision to major debulking surgery up to damage control surgery (DCS). DCS or abbreviated surgery focuses on restoring physiology, at the expense of anatomic continuity while the physical source of infection is managed. In the case of severe IAS, it may include resecting non-viable bowel while deferring anastomosis or stoma creation and/or deferring formal abdominal closure until a later reoperation. This later concept involves the provision of an open abdomen which also mandates an appropriate temporary abdominal closure (TAC). In addition, to be time-saving in the case of DCS, the open abdomen, may better control or remove the source of infection and the associated necrotic/damaged tissues and simplifies further washouts and/or second/multiple revisions including deferred anastomoses [51].

The *mini-invasive non-surgical approach* mainly consists in radiological or endoscopic procedures aiming to drain collections and to let them communicate with the outside of the body and/or hollow viscus cavity. This allows to the infected materials to be evacuated and continue to drain in the subsequent days [52, 53].

### Resuscitation in sepsis

In addition to providing timely SC, patients severe IAS must also be correctly resuscitated. The surviving sepsis campaign launched by the Society of Critical Care Medicine, European Society of Intensive Care Medicine, and the International Sepsis Forum, has formed the standard to which clinicians globally have tried to provide optimal care. The cornerstones of the surviving sepsis bundles are guidelines for the early resuscitation as a medical emergency that should be accomplished early ideally within the first hour, consisting of obtaining lactate and blood cultures, providing broad-spectrum antibiotics, and potentially administering both fluids and vasopressors guided by hemodynamics [54]. However, as outlined below, IAS has never been the focus of these guidelines and the nuances of IAS being a surgical disease speak to the need for surgeons to lead the multidisciplinary team.

### Procedures to restore physiological function

*These procedures and techniques* aim to move beyond simply removing grossly contaminated material to ensure the restoration of optimal physiological function. Technological and scientific advancements allow surgeons and ICU doctors to control the anatomy, the physiology, and the consequence of deranged anatomy and physiology. The control of all these factors are pivotal parts of the SC process. In fact, we should not continue to think to source only with the grossly contaminating material inside the abdomen and consequently SC cannot be considered as the simple removal of that. Modern medicine allows us to see beyond the anatomic/physical aspects of contamination; circulating bacteria, toxins, and mediators are active part of the source of infectious state. Therefore, the authors propose that SC must encompass their removal as well. The comprehensive goals of source control are fully presented in Table 1. They encompass supportive interventions provided both preceding, following, and during actual SC.

## Open abdominal therapy following SC

The term “Open Abdomen” refers to the purposeful leaving of the fascia unapproximated after a laparotomy [21]. In contemporary practice, it also involves leaving the skin open and applying a “temporary abdominal closure (TAC)” dressing to protect the viscera and manage intra-abdominal drainage. It has increasingly been recommended as an option for the most severe cases of IAS without definitive evidence as to its efficacy [55–57]. There are theoretical benefits to this approach including the potential to fundamentally mitigate the inflammatory cascade of biomediators as well as practical benefits in moderating IAH and expediting surgical procedures [58–60]. These benefits must be balanced against the potential increased costs of the technique and potential increased risks of entero-atmospheric fistulae although modern TAC dressings have largely prevented this complication [61]. Given the great potential global benefit, but uncertainty regarding its actual efficacy, the authors strongly recommend all academic institutions support the closed or open after laparotomy for severe complicated intra-abdominal sepsis (COOL Trial) that is currently ongoing to address this question and sponsored by the World Society of Emergency Surgery and the Abdominal Compartment Society [62–65].

### Timings and priorities of SC

The role of SC timing is fundamental but debated. Many papers propose the general principle of “as soon as possible” leaving room for free interpretation of times and methods and techniques. This happens because precise evidence and indications regarding optimal timing of SC in IAI do not exist or vague at minimum. Various published but non-standardized indications exist and SC proposed timings variate from immediately in patients with severe IAI’s, to “as soon as possible,” and up to 7–24 h from diagnosis for IAI without systemic inflammation [18–20, 66, 67].

Surviving sepsis campaign guidelines stated that a “target of 6–12 h after diagnosis should be sufficient for most cases.” They further recommend that any required source control intervention be implemented as soon as medically and logistically practical after the diagnosis is made (BPS) [30]. This recommendation, however, may represent a misunderstanding of surgical sepsis physiopathology in which the larger non-surgical sepsis community does not truly appreciate the timeliness and anatomy of intra-abdominal sepsis. In fact, many consider the Rivers report of 2001 a landmark in sepsis research as “early goal directed therapy” involving early intensive medical resuscitation was associated with dramatic improvements in survival. However, it should be emphasized that this work specifically excluded those in need of emergent surgery for source control. Further data moreover showed no improvements in mortality and complication rate with a goal directed early sepsis management [68].

It has historically been recommended that patients with septic shock from a presumed intra-abdominal source and who are physiologically unstable undergo a period of resuscitation and “optimization” prior to operative SC, with little hard data [69]. Azuhata et al. therefore suggested that any delay may be ill advised, in that if the cause of hemodynamic instability is uncontrolled visceral contamination no amount of resuscitation can help without definitive operative SC. In a prospective evaluation of time to surgical source control, they showed as time to operative source control was critical and highly significant in determining survival. There was 0% survival if surgery was delayed beyond 6 h [70]. Boyd-Carson et al. further corroborated the basis for providing much earlier interventions. Although upper gastro-enteric perforations do not unleash the full burden of fecal peritonitis, it was still found that each additional hour delay to operative SC associated with a 6% increase in mortality [71]. Thus, a patient who arrived in an operating theater within 6 h was 18% more likely to die compared to a patient operated within 1 h [71]. Retrospective data is also consistent with this need for early source control. Rausei et al. [72] noted a 27% mortality versus 9% in those with SC and an open abdomen if treated sooner or later than 6 h, although there were methodological data concerns. Further, it has been quoted that in septic shock a delay to SC greater than 12 h was expected to increase mortality from 25 to 60% compared to a delay of less than 3 h. [72, 73], although again there are severe methodological concerns in quoting an unpublished abstract [74], leaving this as a hypothesis urgently in need further well performed studies.

Thus, the timing of source control depends on the patient’s conditions and the potential evolution of the disease. The authors thus propose three levels of SC urgency:

- *Emergent source control*—in patients with high mortality risk due to a severe physiological derangement caused by the acute disease emergent source control is necessary and must be undertaken as soon as possible after the diagnosis is strongly suspected or established.
- *Urgent source control*—in patient where source control is an essential element of the treatment of infection but is generally assumed that delaying an intervention between 1 and 24 h to improve the clinical condition of patients providing an adequate fluid resuscitation and a broad-spectrum antibiotic therapy.
- *Delayed source control*—in patient where may be appropriate to wait until the demarcation of infectious process, to reduce the risks of collateral surgery damages.

### Patient stratification

The approach to SC must always be related to the disease severity, the source of infection, and to general physiological condition of the patient together with his/her comorbidities. For this reason, it is challenging to accept previous simplistic definitions as being universally valid to describe adequate SC. These multiple variables also mean that tailor-made or individualized approaches to therapy are appropriate making this another example or personalized or precision medicine. Furthermore, the specific cause of infection and its clinical severity will constantly modify the therapeutic strategy. The surgeon should choose the right balance between disease burden, the SC induced physiological derangements, and the potential risk/benefits to the patient [10], keeping in mind that the less robust the patient is, the selected strategy must always “failsafe.” This approach means accepting less risky options such as colostomy rather than an anastomosis in frail patients perceived to be unable to withstand potential complications like anastomotic leaks.

The authors propose that an initial evaluation of the patients’ stratification is a mandatory first step in SC. It should be performed according to their current conditions, their comorbidities and ongoing therapies (i.e., anticoagulants or steroids) together with their immunological state.

Patients can thus be categorized into three classes (Table 2):

- *Class A* Healthy patients with no or well-controlled comorbidities and no immunocompromise, where the infection is the main problem.
- *Class B* Patient with major comorbidities and/or moderate immunocompromise but currently clinically stable, in whom the infection can rapidly worsen the prognosis.
- *Class C* Patients with important comorbidities in advanced stages and/or severe immunocompromise, in which the infection worsens an already severe clinical condition.

**Table 2:** Patients stratification

Patient stratification	
Class A	Healthy patients with no or well-controlled comorbidities and no immunocompromise, where the infection is the main problem
Class B	Patient with major comorbidities and/or moderate immunocompromise but currently clinically stable, in whom the infection can rapidly worsen the prognosis
Class C	Patients with important comorbidities in advanced stages and/or severe immunocompromise, in which the infection worsens an already severe clinical condition

In patients where the immunological conditions are compromised SC may become more complex and require the intervention of other actors besides the surgeon. In fact, complex patients should be managed by multidisciplinary teams to reach the best results. Surgeons, emergency physicians, anesthetist, and infectious disease specialist together with dedicated specialists according to the comorbidities (i.e., hematologists, rheumatologists, oncologists, and/or the teams involved in solid organ transplantation), although we recognize the surgeon as the team leader and ultimate decision maker.

Immunocompromised patients are defined as [75]:

1. Congenital conditions (T- or B-cell defects, macro-phage dysfunctions, often in newborns and children but even in the adult population)
2. Acquired conditions:
  - Infected by human immunodeficiency virus (HIV) who developed acquired immunodeficiency syndrome (AIDS).
  - Hematologic malignancy.
  - Patients affected by intrinsic immune conditions considered immunodeficiency along with one between “solid malignancy or solid organ transplanted patients or inflammatory disease/rheumatologic disease” plus the concurrent assumption of immunomodulatory drugs or chemotherapy.
  - Patients in a physiological or pathologic condition that is accompanied by any degree of immunodeficiency.

Beside the properly defined immunocompromised patients, many other ones present a mix of conditions, surgical risk factors, and physiological status which increase the complications of IAIs and in which the SC must be adapted. We can grossly define a high-risk population based on patient’s conditions (low serum albumin concentration, older age, obesity, smoking, diabetes mellitus, and ischemia secondary to vascular disease or irradiation) or on surgical risk factors (prolonged or delayed/late procedures) [75, 76].

### Definition of adequacy of SC

Underlying causes of IAIs contribute to determine the most adequate SC and the following antibiotic therapy. Adequate SC must encompass whenever necessary all these aspects: gross contamination elimination, solution of the source of the infection, adequate and effective antibiotic therapy and restoring of the physiology by supporting the vital function and by eliminating the circulating mediators and toxins.

As general principle, in uncomplicated IAIs a complete surgical removal and one-shot or short course antibiotic therapy may be sufficient [77].

In complicated IAIs, the surgical strategy, the continuation of the antibiotic therapy, and the physiology supporting strategies must be evaluated continuously based on the intra-abdominal scenario and the patient’s clinical conditions. In this case, a correct assessment of the patient's clinical status must guide the therapeutic choice. For example, healthy patients can sustain SC as in uncomplicated IAIs with greater attention to perioperative antibiotic therapy which must be managed in relation to the clinical state and inflammation indices; on the other hand, patients with already compromised preoperative clinical conditions the choice of the most adequate SC must be more cautious and often decided in a multidisciplinary team with surgeon, anesthetist, and infectious disease specialist.

Surgical effort in pursuing an adequate SC must push on eliminating gross contamination and in solving the source of infection. It may require subsequent procedures in the case of critically ill patients or in the case of complex intra-abdominal situation.

Another important component to emphasize is the bacterial flora causing the infection. Stomach, duodenum, proximal and distal small bowel, colonic perforations, or biliary infections are linked to different bacteria contamination [78]. Lastly the availability of technical expertise and infrastructure at the local institution must be considered for the definition of an adequate SC.

## Antibiotic stewardship

The authors further suggest that all complex and severe IAS be managed in a multidisciplinary fashion including representatives of the Hospital’s Antibiotic Stewardship Committee. While it is imperative to administer the appropriate antibiotics early, it is just as imperative that broad-spectrum antibiotics NOT be continued after they are no longer required and adequate source control has been obtained. This is important for any one patient so as not to induce antibiotic resistance but also to minimize the disruption (if possible) on the human microbiome. Further on a societal basic preservation of antibiotic effectiveness is crucial for all future patients who will require therapy [79, 80].

## Specific intra-abdominal infections (IAI)

### Acute cholecystitis

Many studies over the years have tried to standardize the treatment of acute cholecystitis (AC) [81, 82]. Early treatment (within 7–10 days from symptoms start) laparoscopic/open cholecystectomy is the best strategy in patients fit for surgery.

#### Uncomplicated acute cholecystitis

Class A or B patients with uncomplicated AC: adequate SC represented by cholecystectomy should be performed as urgent procedure with no postoperative antibiotics.

**Fig. 1:**
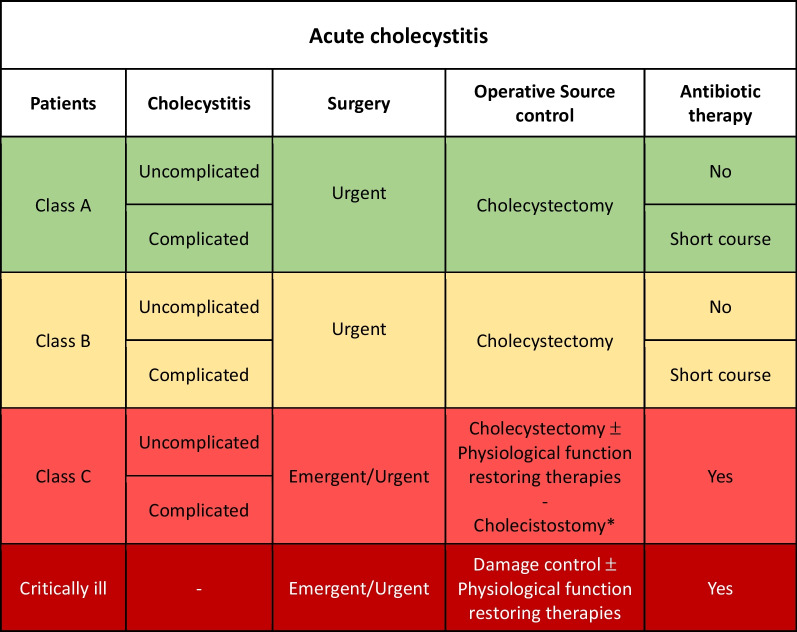
Acute cholecystitis adequate source control indications (*Patients with major comorbidities unfit for surgery and with stable hemodynamic condition may be managed with percutaneous image-guided drainage)

Class C patients with uncomplicated AC: cholecystectomy should be performed as emergent/urgent procedure with postoperative antibiotic therapy (Fig. 1).

#### Complicated acute cholecystitis

Class A or B patients with complicated AC: adequate SC represented by cholecystectomy should be performed as urgent procedure with short course postoperative antibiotic therapy (1–4 days).

Class C patients fit for surgery with complicated AC: cholecystectomy should be performed as emergent procedure with postoperative antibiotic therapy.

In the event of severe hemodynamic instability and diffuse intra-abdominal infection, damage control procedure should be considered independently from the class of patient. Physiological restoring procedures should be associated to the surgical and pharmacological SC.

Cholecystostomy may be an option in critically ill patients with multiple comorbidities and unfit for surgery or patients who do not show clinical improvement after antibiotic therapy for 3–5 days.

### Acute cholangitis

Acute cholangitis is a frequent biliary IAI commonly caused by choledocholithiasis, caused by a combination of biliary obstruction and bacterial growth in bile [83, 84].

**Fig. 2:**
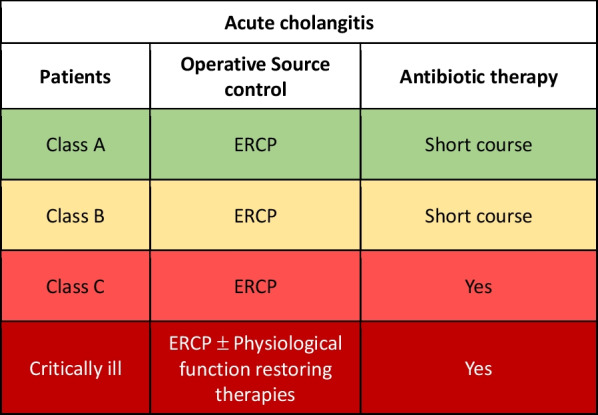
Acute Cholangitis adequate source control indications

SC is based on adequate antimicrobial treatment for 3–5 days and biliary decompression. The type and timing of biliary drainage should be based on the severity of the clinical presentation, and the availability and feasibility of drainage techniques [85, 86] (Fig. 2).

Class A or B patient with acute cholangitis: adequate SC consists in endoscopic retrograde colangio-pancreatography (ERCP) (in the absence of contraindication) and a short course antibiotic therapy.

Class C patients: adequate SC consists in ERCP (in the absence of contraindication) associate to antibiotic therapy; its duration must be identified based on the patient's condition, risk factors for resistant bacteria and its management should be multidisciplinary.

### Acute appendicitis

In acute appendicitis (AA), appendectomy laparoscopic/laparotomic is the gold standard of treatment. About two-thirds of AA are classified as uncomplicated [87].

#### Uncomplicated acute appendicitis

**Fig. 3:**
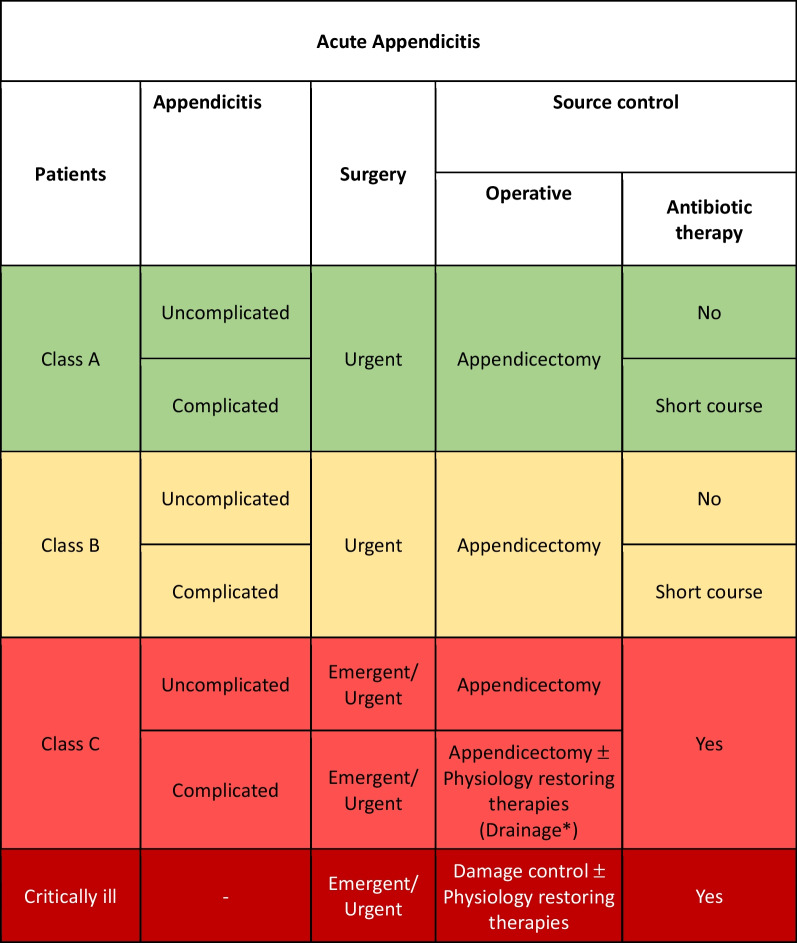
Acute appendicitis adequate source control indications (*Patients with major comorbidities unfit for surgery and peri-appendiceal abscess and with stable hemodynamic condition may be managed with percutaneous image-guided drainage)

Class A or B patients with uncomplicated AA: adequate SC represented by appendectomy should be performed as urgent procedure with no postoperative antibiotics. Conservative treatment with antibiotic therapy and no surgical intervention may be considered in selected cases, but there are studies where it is detected less effective in the long-term due to significant recurrence rates [88, 89] (Fig. 3).

Class C patients with uncomplicated AC: adequate SC represented by appendectomy should be performed as emergent/urgent procedure with postoperative antibiotic therapy. No room for conservative treatment exists in class C patients fit for surgery.

#### Complicated acute appendicitis

Class A or B patients with complicated AA: adequate SC represented by appendectomy should be performed as urgent procedure associated to antibiotic therapy for 4 days, extendable to 7 days if there are signs of infection or systemic disease after surgery [77].

Class C patients with complicated AA: adequate SC represented by appendectomy should be performed as emergent/urgent procedure with postoperative antibiotic therapy. No room for conservative treatment exists in class C patients fit for surgery.

In the event of severe hemodynamic instability and diffuse intra-abdominal infection, damage control procedure should be considered independently from the class of patient. Physiological restoring procedures should be associated to the surgical and pharmacological SC.

Patients with major comorbidities unfit for surgery and peri-appendiceal abscess and with stable hemodynamic condition may be managed with percutaneous image-guided drainage associated to antibiotic therapy [90].

### Acute left colonic diverticulitis

Acute left colonic diverticulitis (ALCD) [78] may be classified into:

Uncomplicated:

- Stage 0 Diverticula, thickening of the colonic wall or increased density of the pericolic fat.

Complicated:

- Stage 1a Pericolic air bubbles or little pericolic fluid without abscess (within 5 cm from inflamed bowel segment).
- Stage 1b Abscess ≤ 4 cm.
- Stage 2a Abscess > 4 cm.
- Stage 2b Distant air (> 5 cm from inflamed bowel segment).
- Stage 3 Diffuse fluid without distant free air (no hole in colon).
- Stage 4 Diffuse fluid with distant free air (persistent hole in colon).

#### Uncomplicated acute left colon diverticulitis

Class A or B patients with uncomplicated ALCD: adequate SC is represented by conservative treatment without antibiotic therapy [91–94].

Class C patients with uncomplicated ALCD and no signs of sepsis: adequate SC is represented by conservative treatment with short course antibiotic therapy (5–7 days) [95].

**Fig. 4:**
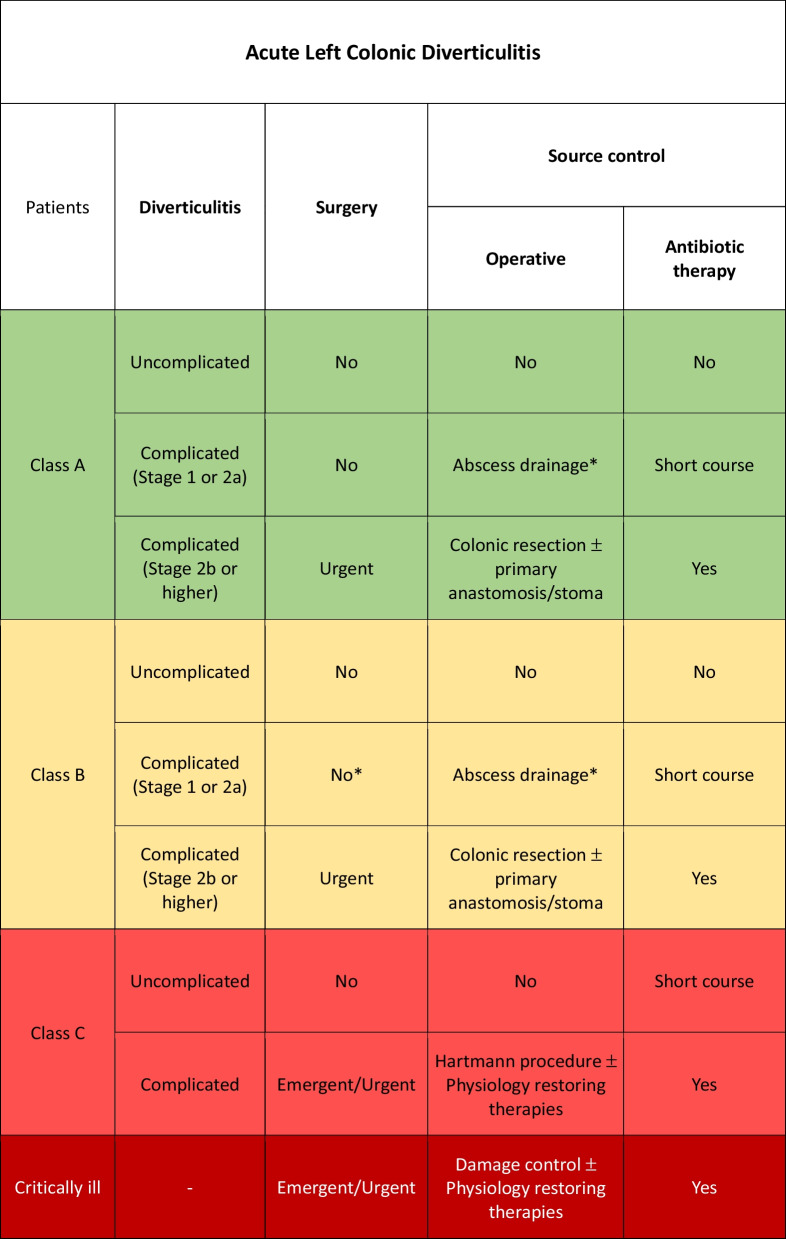
Acute left colonic diverticulitis adequate source control indications (*Percutaneous drainage for abscess larger than 5 cm)

Class C patients with uncomplicated acute diverticulitis and signs of sepsis: initial conservative treatment with antibiotic therapy (Fig. 4).

#### Complicated acute left colon diverticulitis stage 1 or 2a

Class A or B patients with complicated ALCD at stage 1 or 2a: adequate SC consists in antibiotic therapy alone in patients with small diverticular abscesses (< 4–5 cm) while percutaneous drainage combined with antibiotic therapy for 3–5 days is indicated in larger diverticular abscesses [96–98].

#### Complicated acute left colon diverticulitis stage 2b or higher

Surgery is always indicated for adequate SC in patients fit for surgery.

Class A or B patients: adequate SC consists in primary resection and anastomosis with or without a diverting stoma depending on the patient related anastomosis dehiscence risks, associated to antibiotic therapy.

Class C patients: adequate SC consists in Hartmann’s procedure (HP) associated to antibiotic therapy.

Minimally invasive laparoscopic peritoneal lavage and drainage may be considered in class A patients with purulent (but not fecal) peritonitis. This procedure has been proposed in recent years but remains debated [99–101].

In the event of severe hemodynamic instability and diffuse intra-abdominal infection, damage control procedure should be considered independently from the class of patient. Physiological restoring procedures should be associated to the surgical and pharmacological SC [102, 103].

### Acute right colonic diverticulitis

Acute right colonic diverticulitis (ARCD) is less frequent than ALCD but has generally a higher rate of complication that require a surgical treatment [104].

#### Uncomplicated acute right colon diverticulitis

Uncomplicated right colonic diverticulitis should be treated with initial antibiotic therapy [105, 106].

Class A or B patients: adequate SC consists in 5–7 days of antibiotic therapy.

**Fig. 5:**
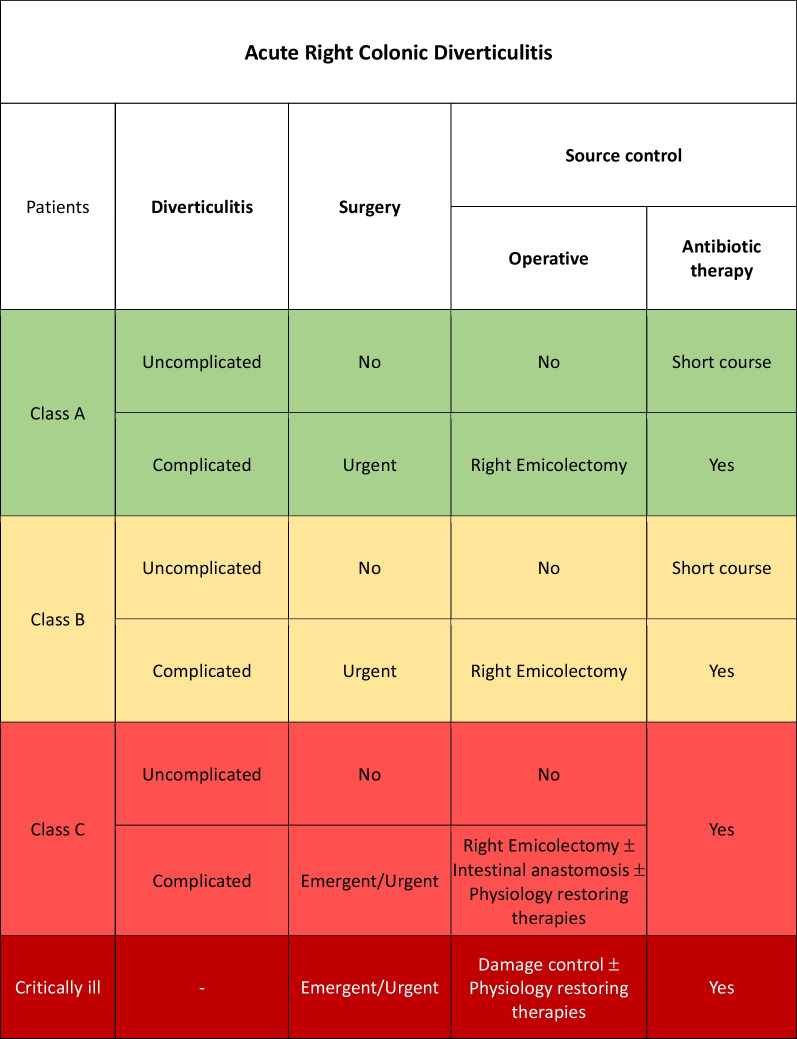
Acute right colonic diverticulitis adequate source control indications

Class C patients: adequate SC consists in antibiotic therapy with a duration that should be discussed according to the clinical condition of patients (Fig. 5).

Once resolved the infectious state right hemicolectomy should be planned after follow-up colonoscopy.

#### Complicated acute right colon diverticulitis

In all patients with complicated ARCD, adequate SC consists in surgical treatment with resection of the inflamed colon and primary anastomosis whenever possible [107] associated to antibiotic therapy. Laparoscopic approach is preferable in experienced centers and in fit patients [108].

In the event of severe hemodynamic instability and diffuse intra-abdominal infection, damage control procedure should be considered independently from the class of patient. Physiological restoring procedures should be associated to the surgical and pharmacological SC.

### Small bowel perforation

In western countries, small bowel perforation is mainly due to unrecognized intestinal ischemia, inflammatory bowel disease (IBD) (i.e., complicated Crohn’s disease) or post-traumatic. In other countries as Asia, Africa, Latin America, the Caribbean, and Oceania small bowel perforation are more often caused by complication of infectious diseases (i.e., typhoid fever) causing a high mortality rate up to 60% [109].

The different causative events may impose differentiated treatment as complicated IBD generally require different management than infectious perforation. Multidisciplinary approach is necessary due to the often-multifactorial causative events (surgeon, ICU doctor, infectious disease specialist, gastroenterologist).

**Fig. 6:**
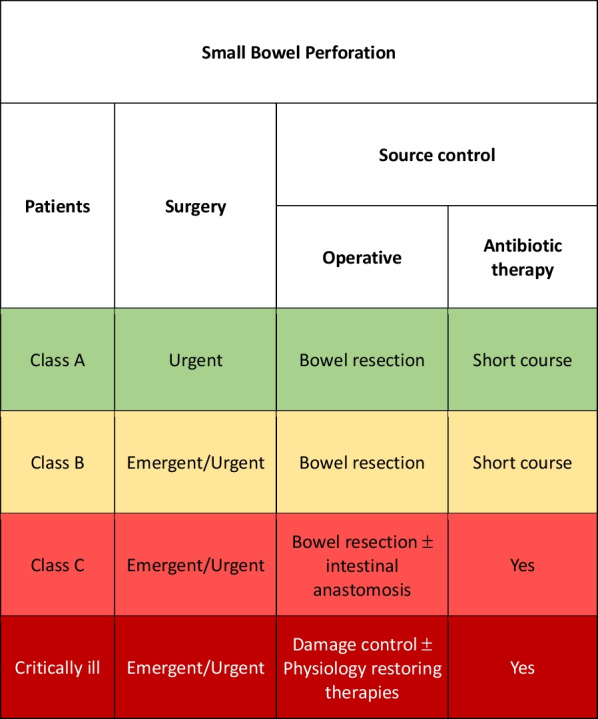
Small bowel perforation adequate source control indications

Class A patient may generally be treated with open or laparoscopic resection with primary anastomosis (whenever possible) and 3–5 days of antibiotics associated to the specific infectious disease treatment. Primary repair should be reserved only in selected patients with minimal peritoneal contamination and a small and single perforation (Fig. 6).

Class B and class C patients must undergo surgery as soon as possible and delayed bowel anastomosis may be considered. Stoma creation or exteriorization of the perforation as a stoma (if distal to the Treitz ligament) should be considered as a valid alternative in the most severe cases [110]. Antibiotic therapy should be continued up to the disappearing of ongoing infection signs.

In the event of severe hemodynamic instability and diffuse intra-abdominal infection, damage control procedure should be considered independently from the class of patient. Physiological restoring procedures should be associated to the surgical and pharmacological SC [111, 112].

### Gastroduodenal ulcer perforation

The most common cause of gastroduodenal ulcer perforation is peptic disease in which Helicobacter pylori is the main etiologic factor. Other causes maybe the use of nonsteroidal anti-inflammatory drugs (NSAIDs), steroids, smoking and a high-salt-content diet. Stress represents an important factor to be considered especially in critically ill patients after surgery or in intensive care units. Conservative management with nil per os, proton pump inhibitors infusion has been described in small and covered perforations with no systemic signs or symptoms of infection [113]. For conservative management the class of patients must be carefully considered.

**Fig. 7:**
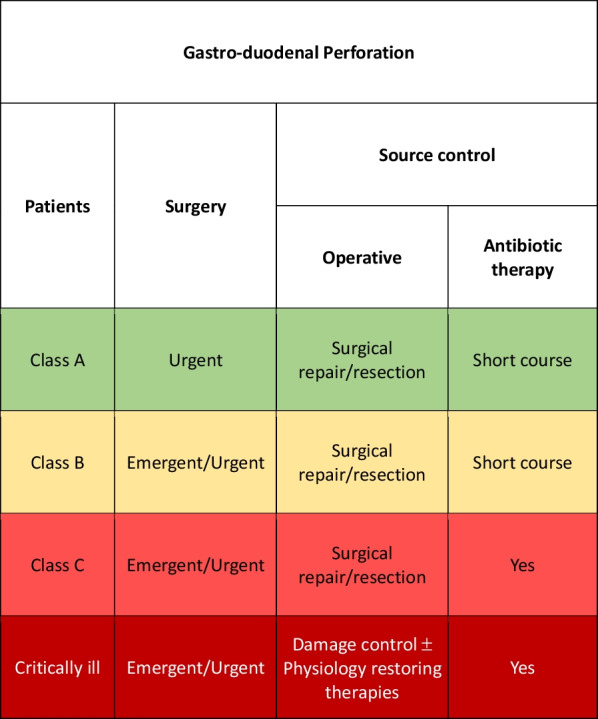
Gastroduodenal perforation adequate source control indications

Class A or B patient should undergo laparoscopic or open simple or double layer suture with or without omental patch. Distal gastrectomy should be reserved in large perforations near the pylorus, in gastric corpus perforations and in suspicious of malignancy [114–116]. Antibiotic therapy is necessary: short course is generally sufficient for class A patients, while in class B the duration should be based on clinical signs of infection (Fig. 7).

Class C patients must be carefully evaluated and operated during or after an adequate resuscitation.

In the event of severe hemodynamic instability and diffuse intra-abdominal infection, damage control procedure should be considered independently from the class of patient. Physiological restoring procedures should be associated to the surgical and pharmacological SC.

### Postoperative peritonitis

Postoperative peritonitis represent a considerable percentage of IAIs which may complicate any surgical intervention with or even without bowel anastomosis. It is a life-threatening condition with potential high mortality rate, because of the diagnosis may not be immediate and may lead to a rapid worsening of the clinical conditions [117, 118] especially in class B or C patients; class A patients, however, should not be underestimated in their possibility of clinical deterioration.

All classes of patient should undergo antibiotic therapy, its duration must be based on the clinical signs of infection and the class of patient [119, 120].

Early re-laparotomy appears to be the most effective means of treating postoperative peritonitis in all classes of patients [121, 122]. Particular attention must be given to critically ill patients with major comorbidities (class B–C), they must be carefully evaluated and operated during or after an adequate resuscitation.

In the event of severe hemodynamic instability and diffuse intra-abdominal infection, damage control procedure should be considered independently from the class of patient. Physiological restoring procedures should be associated to the surgical and pharmacological SC.

### Post-traumatic perforation

Post traumatic perforation is an insidious consequence of trauma, especially in blunt ones. Localized lacerations or transections of the bowel wall, mural and mesenteric hematomas, localized devascularization, and full-thickness contusions may result in immediate or delayed perforation [95]. The treatment is principally early or delayed surgical resection/repair [123, 124].

In Class A patient if the repair is performed very early (within 12 h from trauma) and there are no signs of ongoing infection, perioperative antibiotic therapy is generally sufficient [125].

**Fig. 8:**
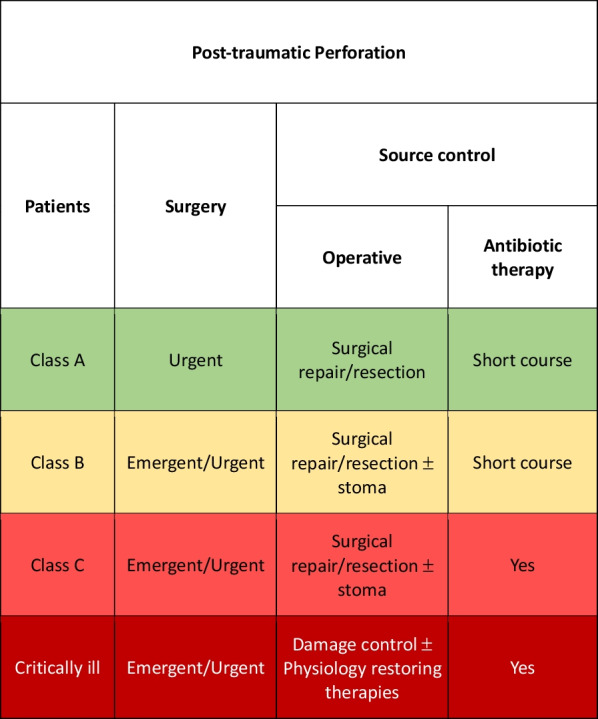
Post-traumatic Perforation adequate source control indications

In class B and C patients after and effective repair antibiotics should be continued until there are no signs of infection [126] (Fig. 8).

In the event of severe hemodynamic instability and diffuse intra-abdominal infection, damage control procedure should be considered independently from the class of patient. Physiological restoring procedures should be associated to the surgical and pharmacological SC.

### Pancreatitis

The management of severe acute pancreatitis (SAP) over the years is changed in favor of a more conservative and minimally invasive approach. Traditionally open surgical debridement was the treatment of choice, but it was burdened with a high failure rate and high mortality rate [127].

SAP associated to necrosis is not a surgical disease at least at the beginning. Its correct management mainly consists in adequate resuscitation and physiological restoring procedures.

Administration of antibiotics, also for prophylactic purposes, in case of pancreatic necrosis without documented infection remains a controversial area but it is conceptually wrong.

Surgery should be delayed in stable patients independently to the class. Contemporary approach to patients with necrotizing pancreatitis and/or infectious pancreatitis could be summarized in the 3Ds: Delay, Drain and Debride [128].

**Fig. 9:**
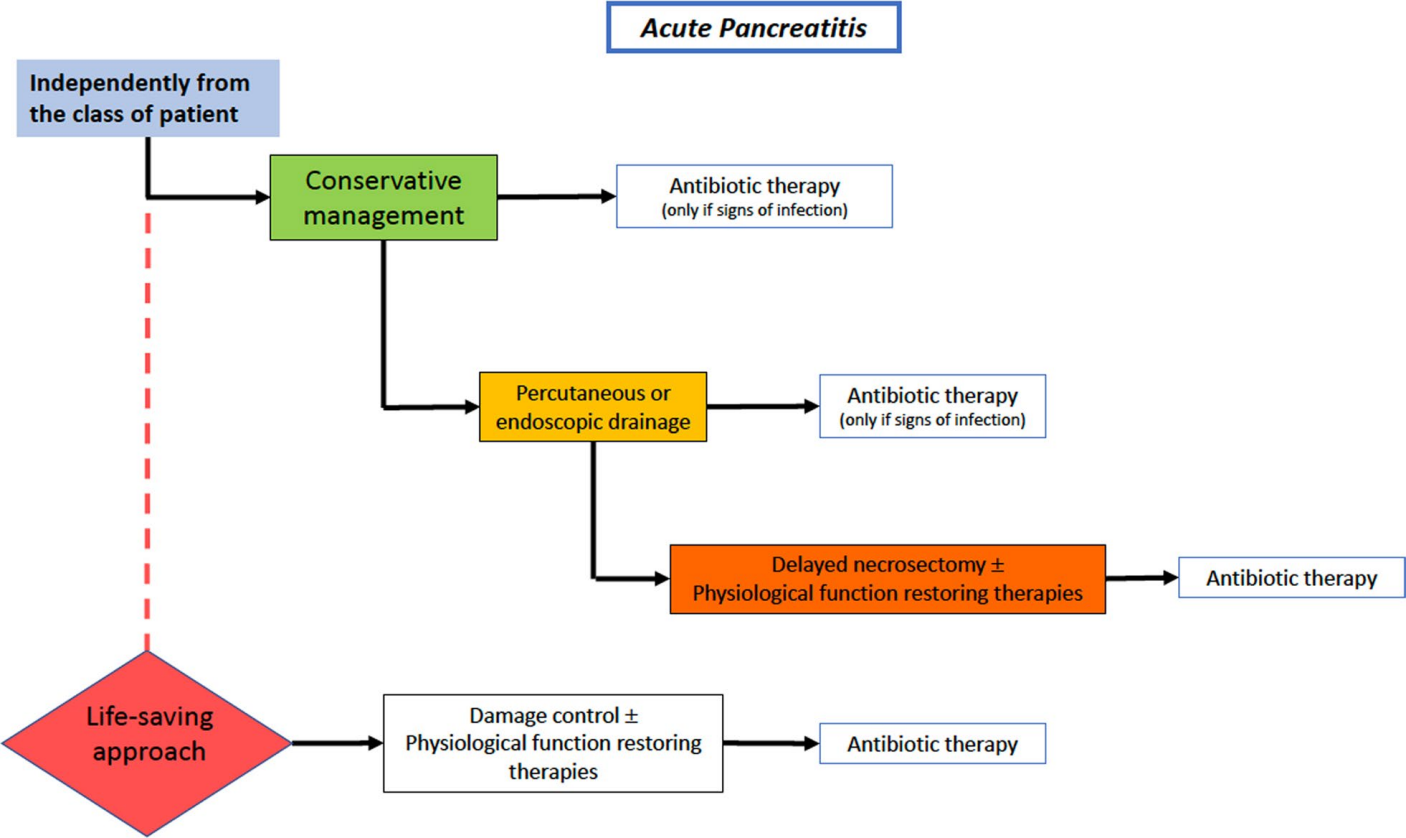
Severe acute pancreatitis adequate source control indications

Independently from the patient’s class, all of them should undergo a conservative approach based on adequate resuscitation and physiological restoring procedures (Fig. 9). Antibiotics therapy should be reserved only in case of signs/symptoms of infection. SC should be implemented in a “step-up” approach in the case of infected pancreatic necrosis. The different steps start from antibiotic therapy up to open surgical debridement associated to damage control procedures, passing through percutaneous or endoscopic drains, minimally invasive retroperitoneal necrosectomy and video-assisted retroperitoneal debridement (VARD) [129] Early open surgery should be considered as a life-saving approach in the cases of rapidly evolving SAP related spies or severe bleeding.

## Necrotizing soft tissue infections

This kind of infections represents the third most frequent cause of severe sepsis and septic shock following pneumonia and IAIs in some series [130]. SC measures in necrotizing soft tissue infections (NSTI) are evident. The specter of diseases that are included in this group can present differently and so categorized, according to causative microorganism, or extension or clinical symptoms. A clinical categorization depending on presence of septic shock and the urgency of requirement for surgical procedures in order to achieve source control has been described [36] with worst outcomes in those with inadequate therapy and sepsis. SC in these infections comprises topical actions, incision and drainage, debridement, up to amputation. Recent recommendations on the approach regarding NSTI states that in uncertain cases time should not be wasted in extensive clinical diagnosis or scoring severity of the patient or hesitating on extension of the first incision [131]. A deep incision up to the fascia should be performed and if NF is diagnosed, radical debridement should be implemented. Independently from the class of patient, prompt and extensive surgery, and a subsequent debridement procedures if necessary to discard ongoing local extension; it must always be associated with broad-spectrum antibiotics [36, 130]. A delayed first surgical intervention (more than 12 h) is associated with higher mortality [132]. Antibiotics should be given as any septic shock patient in the first 6 h, and duration of antibiotic treatment can be between 7–14 days [36, 130].

## Conclusion

Source control adequacy is a complex definition encompassing numerous factors including the causative event, rapidity of diagnosis and responses, source of infection bacteria, local bacterial flora, patient condition, and his/her eventual comorbidities. Adequate source control is no longer only a surgical issue, but requires a multidisciplinary approach to provide the most effective treatment of complicated intra-abdominal infections. The WSES/GAIS/SIS-E/SIS-A has thus proposed a comprehensive working definition of comprehensive source control. The societies hope this approach will enable better clinical care as well as facilitate new research that will both improve patient care and warrant future improvements and revision to the definition.

## Abbreviations

IAIs: Intra-abdominal infections
IAS: Intra-abdominal sepsis
AT: Antibiotic therapy
SC: Source control
IAP: Intra-abdominal pressure
IAH: Intra-abdominal hypertension
ACS: Abdominal compartment syndrome
MSOF: Multi-system organ failure
SCIAS: Severe complicated intra-abdominal sepsis
DCS: Damage control surgery
TAC: Temporary abdominal closure
COOL Trial: Closed or open after laparotomy for severe complicated intra-abdominal sepsis
HIV: Human immunodeficiency virus
AIDS: Acquired immunodeficiency syndrome
AC: Acute cholecystitis
ERCP: Endoscopic retrograde colangio-pancreatography
AA: Acute appendicitis
ALCD: Acute left colonic diverticulitis
ARCD: Acute right colonic diverticulitis
IBD: Inflammatory bowel disease
NSAIDs: Nonsteroidal anti-inflammatory drugs
SAP: Severe acute pancreatitis
VARD: Video-assisted retroperitoneal debridement
NSTI: Necrotizing soft tissue infections
